# Is there a difference between women and men in chronic spontaneous urticaria? A systematic review on gender and sex differences in CSU patients^☆^

**DOI:** 10.1016/j.waojou.2024.100974

**Published:** 2024-10-12

**Authors:** Sarah Preis, Carla Claussen, Stefanie Ziehfreund, Tilo Biedermann, Sophia Horster, Alexander Zink

**Affiliations:** a Technical University of Munich, TUM School of Medicine and Health, Department of Dermatology and Allergy, Munich, Germany; b Institute for Medical Information Processing, Biometry, and Epidemiology, Pettenkofer School of Public Health LMU Munich, Munich, Germany; c University Hospital Munich, Department of Gastroenterology and Hepatology, Munich, Germany

**Keywords:** Gender differences, Chronic spontaneous urticaria

## Abstract

In recent years, there has been a notable surge in interest in gender medicine, with a growing focus on exploring gender and sex differences in skin diseases.

Although it is noticeable in clinical practice that more women than men present with chronic spontaneous urticaria (CSU) in the outpatient setting, there is currently no systematic review available which addresses gender differences in CSU. PubMed Medline, Web of Science, and Cochrane Central Register of Controlled Trials (CENTRAL) were searched until July 2023. English and German randomized controlled trials, prospective and retrospective cohorts, and case-control studies that examined gender and sex differences in CSU were included. Two authors independently screened the reports for eligibility. One extracted all data, the second double-checked and critically appraised the quality and risk of bias of the studies. Twenty-six reports were included. The article reviewed differences in epidemiology, diagnostics, clinical characteristics, treatment, and quality of life in female and male patients. The findings provide limited data for the substantial impact of gender and sex in CSU patients and reveal major gaps in gender-specific care in dermatology which should be narrowed in the upcoming years to optimize patient-centered, individualized, gender-equal healthcare.

**PROSPERO registration:**

CRD42023442958.

## Introduction

Chronic spontaneous urticaria (CSU) is a heterogeneous, inflammatory skin disease clinically characterized by pruritic wheals, angioedema, or both with a duration of more than 6 weeks.^1^^,^^2^ Type I autoimmunity, characterized by “autoallergy” involving IgE against auto allergens, and type IIb autoimmunity (IgG antibodies against the IgE/FcεRI complex) are considered to be implicated in the pathogenesis of the majority of CSU cases.^3^ Both types lead to skin mast cell degranulation, resulting in wheal formation, as well as the release of cytokines, leading to the migration of inflammatory cells such as lymphocytes, neutrophils, basophils, and eosinophils.^4^^,^^5^ Changes in hormonal levels and expression may promote the immunological imbalance responsible for the onset and course of CSU.^6^^,^^7^ CSU has a life-time prevalence of 1–2% and even though no life-threatening consequences exist, CSU is disabling, frustrating, and has a substantial impact on the quality of life.^8^^,^^9^ Although CSU is a clinical diagnosis, laboratory test like autologous serum skin test suggests the presence of autoimmunity if the test results are positive.^2^^,^^9^ The current treatment algorithm for CSU entails treatment escalation from second-generation H1-antihistamins to omalizumab and cyclosporin.^10^

In recent years, there has been a growing understanding of gender and sex differences in medicine, resulting in a rising interest in this subject in clinical research. Both sex, referring to a person's physical characteristics at birth, and gender, encompassing a person's identity, expression, and societal role, affect all most aspects of a disease.^11^ With regard to the immunological effects of urticaria, estrogens can stimulate humoral immunity and antibody syntheses, whilst androgens tend to have an immunosuppressive action.^12^ Mast cells, the main mediator of CSU, are susceptible to sex hormones and express estrogen and androgen receptors.^13^^,^^14^ Whilst estrogens can induce mast cell degranulation, testosterone does not share this effect.^13^^,^^15^ Conditions characterized by hormonal changes, including menstrual cycle, pregnancy, and menopause, seem to have an association with CSU.^7^^,^^16^ So both sex and gender are essential to consider as they may influence different parts of a disease. In other medical disciplines there are comprehensive reviews focusing on gender disparities in epidemiology, clinical characteristics, diagnosis and treatment of diseases.^17^, ^18^, ^19^, ^20^ Until now, dermatology lacks gender-based treatment recommendations primarily because of the absence of specific reviews in this area.

The objective of this systematic review is to examine the current knowledge on sex and gender differences in CSU from various perspectives with special emphasis on epidemiology, clinical characteristics, diagnostics, comorbidities, treatment, and quality of life of female and male CSU patients.

## Methods

### Protocol and guideline statement

This systematic review was registered on the International Prospective Register of Systematic Reviews (PROSPERO) (ID-Number: CRD42023442958). Preference Reporting Items for Systematic Reviews and Meta-Analysis (PRISMA) guidance was followed throughout.^21^

### Search strategy

**Fig. 1:**
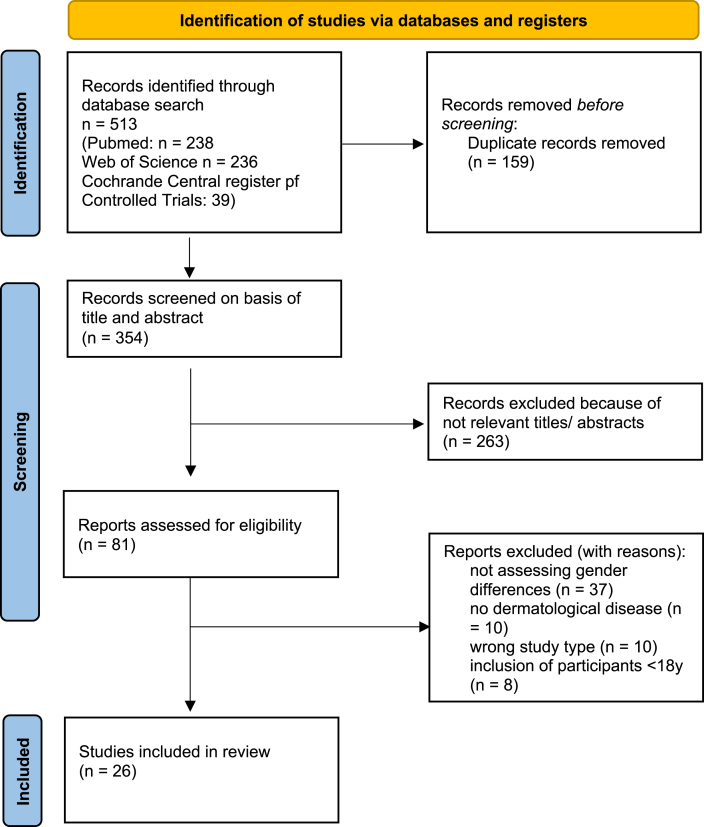
Flow diagram on study selection (following PRISMA 2020).

PubMed Medline, Web of Science, and Cochrane Central Register of Controlled Trials (CENTRAL) were searched using medical subject headings, free-text-terms, study-type filters, and filters for humans and adults only, where appropriate. No time limit has been set for the publications published. The initial search was run on July 19, 2023. The databases searched and the respective search terms are listed in Fig. 1 and Table 2. A manual search was performed in relevant papers for any potentially relevant studies.

### Study selection

Two reviewers (SP and CC) independently conducted searches of all records identified in the search by title and abstract in Endnote. Subsequently, potentially relevant articles were obtained for full-text screening in the subsequent stage. Utilizing a protocol constructed with the PICO (population, intervention, comparison, and outcome) framework, these same reviewers independently evaluated the eligibility of the full-text papers. Any inconsistencies between the reviewers were addressed through discussion, and in case of disagreements, a third author (AZ) was involved to resolve them.

Articles were included if they contained randomized studies (randomized controlled studies (RCTs) or non-randomized studies (prospective and retrospective studies, case-control studies) with relevant outcome data for investigating gender-difference in all aspects of CSU. Case reports, case reviews, reference abstracts/papers, systematic reviews, meta-analysis, and notes were excluded.

### Data selection, data analysis and risk of bias assessment

SP and CC developed a standardized data extraction document using MS Word, encompassing authors’ names, title, study design, country, study setting, study population, participant details, recruitment specifics, inclusion and exclusion criteria, funding and conflicts of interest, outcome data, and risk of bias assessment. Prior to implementation, the data extraction forms underwent pre-testing. Subsequently, SP performed the data extraction, which was then cross-verified by CC to ensure accuracy.

SP performed quality and risk of bias assessments for all included studies using pre-established quality evaluation sheets. The Newcastle-Ottawa Scale (NOS) was employed for cohort and observational studies, while a modified version of the NOS was used for cross-sectional studies.^15^ The grading of evidence in observational studies was independently determined by evaluating three categories: study group selection (4 points), group comparability (2 points), and ascertainment of exposure or outcome of interest (3 points), allowing for a maximum score of 9 (8 for cross-sectional studies).

## Results

### Literature search

In total, 354 non-duplicate articles were identified in the database search, of which 263 were excluded during title and abstract review, and 54 were excluded during full-text review. The final systematic review included 26 studies (Fig. 1).

### Study characteristics

The studies were published between 01/01/2004 and 14/04/2023. Most of the studies (15; 57.7%) were conducted in Europe, followed by Asia (7; 26.9%), South America (2; 7.7%), and the United States (1, 3.8%). An equal proportion of included publications were retrospective, non-interventional, observational studies (10; 37,0%) and cross-sectional, non-interventional studies (10, 37,0%). Three prospective, non-interventional studies and 1 case-control-study were included (Table 1, Supplementary Table 1).

**Table 1:** Studies evaluating gender differences in urticaria.

Key focus	Author Participants analyzed	Design and setting	Outcome	Result: measures of effect	Result summary
Epidemiology	Eun S et al. n = 2980 adults (male: n = 1432)	Retrospective, longitudinal study	*Incidence* Number of patients with CSU/all included patients	Women were at significantly higher risk for the development of chronic urticaria after adjusting for other demographic differences (HR, 1.25; 95% CI, 1.23–1.28) compared with men.	♀
	Gaig P et al. n = 1044 adults (male: n = 408)	Retrospective, population-based study	*Prevalence* Age- and sex- adjusted prevalence of CSU/100,000 adults	The prevalence of CSU is significantly higher in women (0.48%) than in men (0.12%) (OR 3.82; 95% CI: 1.56–9.37).	♀
	Jo YH et al. n = 722 adults (male: n = 308)	Retrospective chart review	*Incidence* Number of patients with CSU/all included patients	Female patients are more affected by CSU.	♀
	Wertenteil S et al. n = 69,570 adults (male: n = 18,560)	Cross-sectional analysis (Multihealth system data analytics and research platform)	*Prevalence* Age- and sex- adjusted prevalence of CSU/100,000 adults	Female patients have a greater prevalence of CSU across all age groups.	♀
Clinical characteristics	Asero R et al. (2009) n = 132 adults (male: n = 44)	Cross-sectional study	*Laboratory parameters* Elevation of D-dimer	Female patients showed higher levels of D-dimer (p < 0.05), which was associated with a longer disease duration (p < 0.01).	♀
	Gregoriou et al. n = 2523 patients (male: n = 614)	Retrospective, observational study	Incidence of etiologic aspects, prognostic factors (comorbidities, disease duration)	Women had a longer disease duration of CSU compared to men (p = 0.001). Women presented more often with urticaria accompanied with angioedema (p = 0.01).	♀
	Savic S et al. n = 252 patients (male: n = 56)	Prospective, non-interventional, multi-center study	Severity of CSU (UAS7), Quality of Life (DLQI)	Female gender was associated with anxiety disorders (p < 0.01) and an increased severity of CSU.	♀
Diagnostics	Aktar S et al. n = 50 adults (male: n = 18)	Case-control study	Positivity of autologous plasma/skin serum test	ASST positivity rates were significantly higher in female subjects (p = 0.001, p = 0.032).	♀
	Kolkhir P et al. n = 1613 adults (male: n = 419)	Retrospective, observational study	Blood count (basophils, eosinophils)	Female patients with CSU had significantly lower blood eosinophile count compared with male patients with CSU (p = 0.001).	♂
	Kurt E et al. n = 55 patients (male: n = 16)	Prospective study	Positivity of autologous skin serum test	ASST positivity rates were significantly higher in female subjects (OR: 3.98, 95% CI: 1.19–13.38).	♀
Comorbidities	Anis O et al.^a^	Cross-sectional, retrospective, population-based study	*Prevalence*	Female gender was associated with the coexistence of these disorders (OR 1.6 95% CI 1.28–2).	♀
	Chen H et al. n = 543 patients (male: n = 165)	Cross-sectional study	*Incidence* Of allergic contact sensitization	Women had more positive reactions toward nickel sulfate (9.26% vs. 3.64%, p = 0.023), while positive reactions to potassium dichromate, benzene mix, and carba mix were more frequent among men than women (14.55% vs. 8.73%, p = 0.042; 6.67% vs. 1.85%, p = 0.004; 18.79% vs. 6.08%, p < 0.001).	♂, ♀
	Confino-Cohen R et al. n = 12,788 patiens (male: n = 4306)	Population-based, retrospective analysis of MHS central data base	*Prevalence of comorbidities*	Female patients with CU had a significantly higher incidence of rheumatoid arthritis (p < 0.0005), Sjögren syndrome (p < 0.0005), celiac disease (p < 0.0005), type I diabetes mellitus (p < 0.0005), and systemic lupus erythematosus (p < 0.0005).	♀
	Chiu H et al. n = 9332 adults (male: n = 3665)	Population-based retrospective cohort study	*Prevalence of comorbidities*	Among women, CSU was significantly associated with atopic dermatitis, allergic rhinitis, autoimmune thyroid diseases, systemic lupus erythematodes, vitiligo, Hennoch-schönlein purpura. Among men, CSU was significantly associated with atopic dermatitis, allergic rhinitis, autoimmune thyroid diseases, Kawasaki disease and inflammatory boewl disease.	♂, ♀
	Ghazanfar M et al. n = 12,185 adults (male: n = 3833)	Retrospective study	*Prevalence of comorbidities*	Female CSU patients have higher odds of thyroiditis and vitiligo compared to males.	♀
	Gupta P et al. n = 481 patients (male: n = 220)	Cross-sectional, observational, single center	*Prevalence of comorbidities*	Males with CSU were more likely to have impaired glucose tolerance compared to controls (p = 0.022), while females with CSU had a higher prevalence of central obesity (p = 0.015).	♂, ♀
	Kolkhir P et al. 2021 n = 1199 patients, (male: n = NA)	Retrospective, observational study	*Prevalence of comorbidities*	Autoimmune comorbidities were reported more frequently in female CSU patients (OR = 2.3, P < 0.001).	♀
Treatment	Grieco T et al. n = 42 patients (male: n = 15)	Prospective, observational, monocentric study	*Relapse* Time to relapse towards omalizumab	Female sex was associated to a significantly higher frequency of recurrence (77.4% in respect to male sex 36.4%, p = 0.024).	♂
	Kocaturk E et al. n = 110 adults (male: n = 36)	Retrospective, observational study	*Treatment response* OS of treatment response by UCT >12 = under control	Female gender was more frequent in non-responders towards CsA and Omalizumab (p = 0.017).	♀
	Tagka A et al. n = 108 adults (male: n = 25)	Prospective, observational study	*Treatment response* Change from baseline in UAS	Females manifested significantly higher CSU scores as compared to males. Females manifested lower levels of CSU percentage change (p = 0.03).	♀
	Sirufo MM et al. n = 42 adults (male: n = 16)	Prospective, non-interventional, single-center study	*Treatment response* Change from baseline in DQLI, UAS and CU-Q2oL	No gender differences in treatment response were observed. Male gender predicted higher rates of recurrence (recovery rate: 25% vs. 84.6%).	♂, ♀
	Sommer R et al. N = 103 adults (male: n = 29)	Cross-sectional, non-interventional study	*Treatment burden* DLQI, CU-Q2oL, Patient Needs Questionnaire	In the PNQ, women rated four items higher compared to men.	♀
Quality of Life	De Ue et al. n = 62 adults (male: n = 17)	Cross-sectional, non-interventional study	*Quality of life* Differences in DLQI, SF-36	Quality of life was found to be more affected in women.	♀
	Erol K et al. n = 103 adults (male: n = 27)	Cross-sectional, non-interventional study	*Quality of life* Differences in DLQI, HADS, FSS	Female patients showed more fatigue than males and had significantly higher scores pf FSS (p = 0.025).	♀
	Sanchez-Diaz et al. n = 77 adults (male: n = 22)	Cross-sectional, non-interventional study	*Quality of life* Differences in DLQI, CU-Q2oL, International Index of Erectile Function (IIEF-5), Female Sexual Function Index (FSFI-6), NRS for sexual impairment	Female sex was significantly associated with poorer general dermatologic and urticaria-specific quality of life (p < 0.05). Female sexual dysfunction was associated with an increase in the risk for anxiety by 85% (OR: 1.85, CI: 1.03–3.30, p = 0.002), depression by 90% (OR: 1.90, CI: 1.02–3.88, p = 0.04) and sleep disturbances (p < 0.001).	♀
	Silvares M et al. n = 100 adults (male: n = 14)	Cross-sectional, non-interventional study	*Quality of life* Differences in DLQI	Female patients reported greater impact on cloth- ing, while male patients reported treatment interference with work and study (p < 0.05).	♂, ♀

*♂*, *♀* indicates which gender was more affected/presents the worser outcome. ASST: autologous serum skin test; CI: confidence interval; CsA: cyclosporin A; CSU: chronic spontaneous urticara; CU-Q2oL: chronic urticaria quality of life test; DLQI: Dermatology Life Quality Index; FSFI-6: Female Sexual Function Index; FSS: fatigue severity scale; HADS: hospital anxiety and depression scale; IIEF-5: International Index of Erectile Function; OR: odds ratio; SF-36: short form 36; UAS7: urticaria severity score; UCT: urticaria control test.

a Only abstract available

**Table 2:** Search strategy.

Population	
Chronic urticaria (3964)	"chronic urticaria"[MeSH Terms] OR ("chronic"[All Fields] AND “urticaria"[All Fields]) OR "chronic urticaria"[All Fields]
AND	
Gender	"gender identity"[MeSH Terms] OR ("gender"[All Fields] AND "identity"[All Fields]) OR "gender identity"[All Fields] OR "gendered"[All Fields] OR "gender s"[All Fields] OR "gendering"[All Fields] OR "genderized"[All Fields] OR "genders"[All Fields] OR "sex"[MeSH Terms] OR "sex"[All Fields] OR "gender"[All Fields]
AND NOT	
Systematic review, review, case report, letter	

SEARCH TERM PUB MED: (("chronic urticaria"[MeSH Terms] OR ("chronic"[All Fields] AND "urticaria"[All Fields]) OR "chronic urticaria"[All Fields]) AND ("sex"[MeSH Terms] OR "sex"[All Fields] OR ("gender identity"[MeSH Terms] OR ("gender"[All Fields] AND "identity"[All Fields]) OR "gender identity"[All Fields] OR "gendered"[All Fields] OR "gender s"[All Fields] OR "gendering"[All Fields] OR "genderized"[All Fields] OR "genders"[All Fields] OR "sex"[MeSH Terms] OR "sex"[All Fields] OR "gender"[All Fields]))) AND ((humans[Filter]) AND (english[Filter] OR german[Filter]))

### Risk of bias

In all included observational studies, the risk of bias was low and the overall quality of all studies high (Table 3).

**Table 3:** RoB assessment of the included studies

Study	Representativeness of the exposed cohort	Selection of the non-exposed cohort	Ascertainment of exposure	Demonstration that outcome of interest was not present at start of the study	Comparability of cohorts	Assessment of outcome	Was follow up long enough for outcomes to occur	Adequacy	Total
Eun S et al. (2019)	+	–	+	+	++	+	+	+	8
Gaig P et al. (2004)	+	–	–	+	++	–	+	+	6
Jo YH et al. (2022)	+	–	+	+	++	+	+	+	8
Wertenteil S et al. (2019)	+	–	+	+	++	+	+	+	8
Asero R et al. (2020)	+	+	+	+	++	+	+	+	9
Gregoriou et al. (2009)	+	–	+	+	++	+	+	+	8
Savic S et al. (2020)	+	–	+	+	+	+	+	–	7
Aktar S. et al. (2015)	+	+	+	+	++	+	+	+	9
Kolkhir P et al. (2020)	+	–	+	+	+	+	+	+	7
Kurt E et al. (2011)	+	–	+	+	+	+	+	+	7
Anis O et al. (2023)	+	+	+	+	++	+	+	+	9
Chen H et al. (2016)	+	–	+	+	+	+	+	+	7
Chiu HY et al. (2018)	+	–	+	+	++	+	+	+	8
Confino-Cohen R et al. (2012)	+	+	+	+	++	+	+	+	9
Ghazanfar M et al. (2020)	+	+	+	+	++	+	+	+	9
Gupta P et al. (2023)	+	–	+	+	++	+	+	+	8
Kolkhir P et al. (2021)	+	–	+	+	++	+	+	+	8
Kokaturk E et al. (2021)	+	–	+	+	+	+	+	+	7
Grieco T et al. (2020)	+	–	+	+	++	+	+	+	8
Tagka A et al. (2021)	+	–	+	+	++	+	+	+	8
Sirufo M et al. (2021)	+	–	+	+	++	+	+	+	8
Sommer R. et al. (2020)	+	–	+	+	++	+	+	+	8
De Ue et al. (2011)	+	–	+	+	++	+	+	+	8
Erol K et al. (2020)	+	–	+	+	++	+	+	+	8
Sanchez-Diaz M et al. (2023)	+	–	+	+	++	+	+	+	8
Silvares M et al. (2011)	+	–	+	+	++	+	+	+	8

### Epidemiology

All identified studies reported higher incidence and prevalence rates for CSU in female patients compared to males.^22^, ^23^, ^24^, ^25^ In a population-based study in Spain, the comparison between sexes disclosed a clear-cut female preponderance with an odds ratio (OR) of 3.82.^23^ Similar results were seen in the United States, where the standardized prevalence of CSU was 2-fold greater in women (309.3 [95% CI 306.6- 312.1] cases/100,000 adults) than men (145.5 [95% CI 143.4–147.7] cases/100,000 adults, p < 0.001).^25^ Strikingly, women aged 40–49 years have the highest prevalence rate of CSU, whilst the age of onset followed the same profile in women as in men.^23^, ^24^, ^25^ Regarding new onset of CSU, the highest female predominance could be seen for females aged 20–44 and 45–64 years.^22^

### Clinical characteristics

Gregoriou et al described that females had a longer disease duration of CSU (33.3 ± 1.4 months vs 25.7 ± 1.9 months; p = 0.001) and a higher rate of angioedema accompanying CSU (p = 0.01).^26^ Likewise, a higher severity of CSU showed an association with female sex.^27^^,^^28^ Regarding the severity of CSU, males manifested a mean Urticaria Activity Score Over 7 Days (UAS7) 29.26 ± 9.74 at diagnosis, whereas females manifested a mean UAS7 score 34.19 ± 8.96 at diagnosis (p = 0.024).^28^

### Diagnostics

A positive response to autologous serum skin test (ASST) was significantly associated with diagnosis of CSU (OR: 3.13, 95% CI: 1.25–7.87) and with female gender (OR: 3.98, 95% CI: 1.19–13.38).^29^ Female CSU patients had higher ASST positivity rates.^30^ Regarding the peripheral blood count of CSU patients, female patients had significantly lower blood eosinophile count compared with male patients with CSU (p = 0.001).^31^ There was an association between eosinopenia and ASST positivity.^31^ When examining laboratory parameters, female CSU patients showed a higher prevalence of elevated D-dimers (p < 0.05), which are accompanied by a significantly longer disease duration than patients showing normal D-dimer levels (mean 64.6 months [range 2–600 months; median 21 months] vs mean 28.6 months [range 2–500 months; median 6 months] p < 0.01).^32^

### Comorbidities

Female and male patients with CSU showed different clusters of comorbidities.^1^^,^^33^, ^34^, ^35^, ^36^, ^37^, ^38^ Autoimmune thyroid diseases, atopic dermatitis, and allergic rhinitis were associated with CSU in both female and male patients.^33^^,^^36^ Other autoimmune comorbidities were reported more frequently in female CSU patients (OR = 2.3, p < 0.001) and included thyroiditis, vitiligo, systemic lupus erythematosus, rheumatoid arthritis, Sjögren syndrome, diabetes mellitus I, and Hennoch-Schonlein purpura.^33^^,^^36^^,^^38^ Chiu et al. reported higher rates of Kawasaki disease (OR 2.99) and inflammatory bowel disease (OR: 1.41) in male, but not in female CSU patients.^33^ Regarding metabolic syndrome, males with CSU were more likely to have an impaired glucose tolerance (p = 0.022), while females with CSU had a higher prevalence of central obesity (p = 0.015).^37^ Anis et al. investigated the correlation of CSU and interstitial cystitis/bladder pain and reported an association of female gender with these disorders (OR: 1.6; 95% CI: 1.28–2.0).^34^ Gender differences can be furthermore found in allergic contact sensitization, where positive reactions to nickel sulfate were more common among women than men (p = 0.023); men reacted more frequently towards potassium dichromate, benzene, and carba mix.^35^

### Treatment

Seven included studies assessed gender differences in the treatment of CSU.^28^^,^^38^, ^39^, ^40^, ^41^, ^42^, ^43^ These showed partially contradictory results. Sirufo et al examined 42 patients treated with omalizumab.^43^ All patients, regardless of sex, age, or any other factor achieved clinical remission of the disease after the first 3 doses with a reduction of disease activity indices and impact on the quality of life.^43^ Whilst they described females to have a more durable remission, Grieco et al. associated female sex to a significantly higher frequency of recurrence (77.4% in respect to male sex 36.4%, p = 0.024).^39^ When considering CSU patients who fail in therapy with both omalizumab and cyclosporin A (CyA), the majority of these patients were female (21; 87.5% vs. 53; 61.6%; p = 0.017).^40^

### Quality of life

Sommer et al assessed patient burden and needs in treatment of CSU patients using the Patient Need Questionnaire (PNQ, 5-Point-Lickert-Scale; 0 = “not important at all”, 4 = “very important”).^44^ Differences in the patients’ needs were found for the needs “to be able to accept oneself” PNQ score: 2.83 for women vs. 1.85 for men, p = 0.017), “to find a clear diagnosis and therapy” (3.86 for women vs. 3.43 for men, p = 0.007), “to be healed of all skin defects” (3.78 for women vs. 3.41 for men, p = 0.005), and “to be less helpless against the disease” (3.66 for women vs. 3.39 for men, p = 0.021). The overall quality of life (QoL) was found to be more affected in women, with statistically significant differences compared to men in the daily activities domain of the Dermatology Life Quality Index (DLQI; p = 0.003) and in the vitality (p = 0.038), role-emotional (p = 0.018) and mental health (p = 0.020) domains.^45^ Silvares et al described that the mean total DLQI as “severe impairment of quality in life” was classified by a female predominance (6:1).^46^ Assessing chronic fatigue, a disease with a significant impact on QoL in patients, 55.3% of female patients with CSU reported fatigue, whereas only 29.6% of male CSU patients were affected (p = 0.022).^47^ Sanchez-Diaz et al assessed the prevalence and potential impact of sexual dysfunction in CSU patients.^48^ Female patients had a poorer QoL and the presence of sexual dysfunction was strongly related to a low life quality and increased the risk for anxiety by 85% (OR: 1.85, CI: 1.03–3.30, p = 0.002), depression by 90% (OR: 1.90, CI: 1.02–3.88, p = 0.04) and sleep disturbances (p < 0.001).^48^ In contrast, male sexual dysfunction was not related to quality of life, anxiety, depression, or sleep disturbances.^48^

## Discussion

Gender and sex disparities are prominent across various facets of CSU, encompassing epidemiology, clinical manifestations, diagnostic indicators, comorbidities, treatment responses, and their consequent implications for quality of life. Women, particularly those aged 40–49, experience higher incidence and severity, leading to extended disease durations and a more significant impact on their overall well-being compared to men.

All identified studies reported a higher prevalence and incidence of CSU in females.^22^, ^23^, ^24^, ^25^ This difference is presumed to be due to sex hormones and autoimmunity, which influence the pathogenesis of the disease.^23^^,^^25^^,^^49^ Estrogen, which is enhanced in the fertile phase of women, is believed to increase humoral immunity and antibody synthesis, whilst estradiol leads to mast cell activation.^49^^,^^50^ Regarding new onset of CSU, the highest female predominance could be seen for females aged 20–44 and 45–64 years.^22^ In the other age groups, men and women were comparable.^22^ The peak timepoints of CSU in women correlate with hormonal fluctuations of women's lives, with an age of 20–44 years as a time of pregnancy and lactation period and 45–64 as hormonal upheaval of the menopause.^7^^,^^51^ As no such highly hormone-active phases occur in men's lives, the most prominent difference between genders concerning age groups can be elucidated in this manner.

Mechanisms triggering the pathogenesis of CSU have been identified as type I auto allergic (association with IgE antibodies against autoantigens) and type IIb autoimmune (driven by autoantibodies FceR1 and/or IgE).^52^ ASST positivity is included among others in the diagnostic criteria for type IIb CSU.^52^ Type IIb CSU patients are often non-responders to omalizumab therapy.^52^ Therefore, ASST as a screening test could find its way into clinical practice to endotype patients with CSU, regardless of gender, for better clinical management especially beyond anti-IgE-therapy.^52^^,^^53^ Another parameter to identify treatment response was eosinopenia, described by Kolkhir et al.^31^ Eosinopenia tends to worsen the treatment response in CSU patients and was found to be predominantly in females. As both parameters have a female predominance, one could think of a female-specific blood test to identify females at risk for treatment failure to be able to offer personalized care.

Female sex was regarded as a predictor for non-response towards omalizumab and CsA therapy.

The finding regarding the gender-specific therapy response is unsurprising, given that the systematic review has indicated a correlation between high autoimmune activities, linked to increased instances of CSU type IIb, along with eosinopenia, and a diminished response to omalizumab. Additionally, these 2 features are more commonly found in women. It would be interesting to observe the extent to which women and men without underlying autoimmune conditions differ in their response to therapy. Currently, however, there is no available data on this matter, but this should prompt the conducting of medication studies that are gender/sex- and subgroup-differentiated. For other skin diseases, as psoriasis, it was described that women suffer from significantly more side effects compared to males which leads to a discontinuation of the medication.^54^^,^^55^ If medication is administered for too short a period of time, it is often not effective. However, current data on omalizumab are lacking to confirm this hypothesis.

When comparing comorbidities, it is known that autoimmunity and autoimmune processes are thought to be a frequent underlying cause of CSU, which are more common in female patients.^1^^,^^22^^,^^33^^,^^34^^,^^36^^,^^38^ When treating the underlying autoimmune condition, there is often an improvement in CSU as well. Here, gender differences in autoimmune conditions emerge as a problem independent of CSU. We are aware that women with autoimmune conditions are often inadequately treated compared to men. In many autoimmune diseases, for example rheumatoid arthritis, women have lower remission and treatment response rates compared to men and higher chances of discontinuation of a drug.^54^ From a gender perspective, the initial focus should be on establishing current gender-specific guidelines for diagnosing and treating underlying comorbidities. On one hand, a gender-specific screening method might be indicated for women to detect autoimmune diseases early. On the other hand, clinical manifestations of many autoimmune conditions differ in female and male patients, potentially necessitating the establishment of differentiated screening parameters.^55^, ^56^, ^57^ This highlights the well-known issues of gender medicine: female participants are underrepresented in disease and drug studies, resulting in mostly inadequate or incorrect screening methods, diagnostic pathways, and medication for female patients. If female patients were adequately diagnosed and treated, this could potentially improve the prognosis of their CSU.

In terms of treatment needs, findings indicate a difference between female and male CSU patients, as most patients' need were rated as more relevant by women than by men.^44^ The dominantly female-rated patient needs were to “be healed from all skin defects”, “be able to accept oneself” and “be less helpless against the disease”.^44^ These needs are related to self-image, visibility of symptoms, and the need for an internal health locus of control, attributes of self, and understanding of illness in women.^58^ Gender differences regarding treatment expectations were seen for other skin diseases as well, in psoriasis the greatest differences were seen in the feeling of depression, sleep quality, and everyday productivity.^59^ For this, various hypotheses can be proposed: on one hand, the patient need “be healed from all skin defects” might indicate that women in our society are more often reduced to their appearance and therefore experience more external pressure; on the other hand, there is also a different understanding of illness between men and women in our society, which could lead to these items being of greater importance to women.

Skin diseases like urticaria potentially exert a negative impact on patients' quality of life.^60^ The degree of psychological stress, embarrassment and stigmatization may not correlate with the clinical severity of the disease and may result in a significant decrease in social and emotional well-being.^45^^,^^60^ When taking into account women's’ higher disease severity levels, longer disease durations and worse treatment outcome women suffer the impact of chronic urticaria more than men. The DLQI includes questions on self-care, choice of clothing, and taking care of oneself or the house. Possible explanations include those domains, that are affected in the DLQI in females more than in males, may involve situations that lead to greater repercussions on women about appearance or maintenance of the house.

In dermatology, as in other medical disciplines, we do not know whether differences in diseases, blood values, behaviors, etc., stem from sex or gender, as these two areas are strongly interconnected and due to millennia-old gender roles, are difficult to separate. Typically, biologically measurable parameters such as hormones and blood values are more often associated with sex, while sensations like QoL are linked to social gender. Corresponding to the much higher societal expectations regarding beauty for women, the perceived impact of skin diseases would then be higher in individuals identifying with the female gender role. This can only be changed through a rethinking of the female gender role in society. CSU had greater effects on the mental health and vitality of female patients, indicating a stronger influence on women because the self-perception of being "ugly" affects a woman in every aspect of life more significantly than men. In our society, women are more profoundly destabilized in every aspect of life by visually "disturbing/imperfect" conditions because they have learned to place greater value on others' opinions. Female CSU patients consequently need better support in coping with the condition and, simultaneously, early screening tools to detect and intervene in mental distress at an early stage.

## Strengths and limitations

This is the first systematic synthesis of data on gender and sex differences in CSU. The use of standardized review techniques helped to minimize the risk of bias. Furthermore, we assessed the quality and risk of bias of each study using the NOS. The main limitations of observational studies are differences in design, outcomes, and a heterogenous collective of participants. Only studies focusing on the subjective well-being and QoL of patients took into account biological and sociocultural gender perspectives in their discussions. Variances in self-care and clothing habits were attributed to social constructs and societal expectations within which the patients were situated.^45^, ^46^, ^47^, ^48^ All other studies either addressed biological gender specifically or regarded biological and social gender as a unified entity. No data was provided in any of the included studies regarding the amount of patients having chosen a gender role that was divergent from their biological sex.

## Conclusion

The results of the systematic review indicate that there are only a few studies addressing gender and sex differences in CSU and our work clearly demonstrates once again that due to research being predominantly centered on the male gender for a long time, numerous gaps exist regarding why women and men differ in diseases. It is a fact and should not be further ignored in the era of personalized medicine that women and men exhibit different progressions in illnesses, and both genders deserve their own diagnostics and treatments. Not only in dermatology, but in every single specialty.

## Abbreviations

ASST, autologous serum skin test; CI, confidence interval; CSU, chronic spontaneous urticaria; CyA, cyclosporine A; DLQI, Dermatology Life Quality Index; IgE, immunoglobuline E; IgG, immunoglobuline G; NOS, Newcastle-Ottawa Scale; PICO, population, intervention, comparison, outcome; PNQ, Patient Need Questionnaire; PROSPERO, Prospective Register of Systematic Reviews; QoL, quality of life; RCT, randomized controlled studies.

## Funding

The study was funded by the Department of Dermatology and Allergy, School of Medicine and Health, Technical University of Munich, Munich, Germany.

## Availabilty of data and materials

Data sharing is not applicable to this article as no data sets were generated or analyzed during the current study.

## Author contributions

The authors confirm contribution to the paper as follows: study conception and design: SP, SZ; data collection: SP, CC; analysis and interpretation of results: SP, AZ; draft manuscript preparation: SP; manuscript review and proof-reading: AZ, SH. All authors reviewed the results and approved the final version of the manuscript.

## Ethics statement

Institutional review board approval and informed consent were not applicable for this study.

## Consent for publication

The participants signed consent regarding publishing their data.

## Authors’ consent for publication

All authors approved the manuscript and gave their consent for submission and publication.

## Declaration of competing interest

SP received speaker's honoraria from Janssen, Novartis, Abbvie. CC and SH have no conflict of interest to declare. SZ has no conflict of interest to declare. TB gave advice to or received a honorarium for talks or research grants from the following companies: ALK-Abelló, Janssen, Meda, Novartis, Phadia Thermo Fisher, Sanofi, and Celgene. AZ has been an advisor and/or received speaker's honoraria and/or received grants and/or participated in clinical trials from/of the following companies: AbbVie, ALK Abello, Almirall, Amgen, Beiersdorf Dermo Medical, Bencard Allergie, BMS, Celgene, Eli Lilly, GSK, Incyte, Janssen Cilag, Leo Pharma, Miltenyi Biotec, MSD, Novartis, Pfizer, Sanofi-Aventis, Takeda Pharma, Thermo Fisher Scientific Phadia, UCB.
