# Supplementary material for: Is there a difference between women and men in chronic spontaneous urticaria? A systematic review on gender and sex differences in CSU patients
Source: World Allergy Organ J. 2024 Oct 12;17(11):100974. doi: 10.1016/j.waojou.2024.100974 (PMC11838081; doi:10.1016/j.waojou.2024.100974)
Supplement: Multimedia component 1 [file mmc1.pdf]

Supplementary Table 1: Studies evaluating gender differences in urticaria

Study details	Population details	Key focus	Outcome, Timepoint, participants included in the analysis	Results
Eun S et al. 2019<br><br>**Study name:** NA<br><br>**Design and setting:** Retrospective, longitudinal study<br><br>**Country:** South Korea	**Sample:** 1027620 patients<br>female: n = 508863<br>male: n = 518757,<br>mean age: NA<br><br>**Diagnostic criteria:** CSU (ICD-code)	Epidemiology (Incidence)	**Outcome:** Incidence of CSU in the National Health Insurance Service-National Sample Cohort<br><br>**Timepoint**: 2004-2013<br><br>**Participants included:** 2980 patients<br>female: n = 1548,<br>male: n = 1432	Females have a higher risk of onset of CSU (HR: 1.25, 95% CI: 1.23-1.28).
Gaig P et al. 2004<br><br>**Study name:** NA<br><br>**Design and setting:** Retrospective, population-based study<br><br>**Country:** Spain	**Sample:** 5003 patients<br>female: n = 2567<br>male: n = 2436,<br>mean age: NA<br><br>**Diagnostic criteria:** CSU (ICD-code)	Epidemiology (Prevalence)	**Outcome:** Prevalence of CSU in a demographically heterogenous population-based sample<br><br>**Timepoint**: NA<br><br>**Participants included:** 1044 patients<br>female: n = 636,<br>male: n = 408	The prevalence of CSU is significantly higher in women (0.48%) than in men (0.12%) (OR 3.82; 95% CI:1.56-9.37). The age of onset followed the same profile in men as in women.
Jo YH et al. 2022<br><br>**Study name:** NA<br><br>**Design and setting:** Retrospective chart review<br><br>**Country:** South Korea	**Sample:** 772 patients<br>female: n = 464,<br>male: n = 308,<br>mean age: 43.1 ± 14.6<br><br>**Diagnostic criteria:** CSU (ICD-code)	Epidemiology (incidence)	**Outcome:** Incidence of CSU<br><br>**Timepoint**: 01/2011-12/2018<br><br>**Participants included:** 722 patients<br>female: n = 464,<br>male: n = 308	The female sex ratio was higher, especially for patients over the age of 40.
Wertenteil S et al. 2019<br><br>**Study name:** NA<br><br>**Design and setting:** Cross-sectional analysis (Multihealth	**Sample:** 69570 patients<br>female: n = 51010,<br>male: n = 18560,<br>mean age: 18-29: n = 11,690, 30-39 n = 10,540, 40-49 n =	Epidemiology (Prevalence)	**Outcome:** Prevalence of CSU in a demographically heterogenous population-based sample<br><br>**Timepoint**: 2012-2017<br><br>**Participants included:** 69570 patients<br>female: n = 51010,	The standardized prevalence of CSU was 2-fold greater in women (309.3 [95% CI 306.6-312.1] cases/100,000 adults) than men (145.5 [95% CI 143.4-147.7] cases/100,000 adults, $p < 0.001$).<br><br>Female patients had a greater prevalence of CSU across all age groups. The highest prevalence of CSU was observed among women aged 40-49

system data analytics and research platform)<br><br>**Country:** USA	11,880, 50-59 n = 12,560,<br>>60 n = 22,900<br><br>**Diagnostic criteria:** CSU (ICD-code)		male: n = 18560	years (364.0 [95% CI 356.6-371.5] cases per 100,000 adults).
Asero R et al. 2019<br><br>**Study name:** NA<br><br>**Design and setting:** Retrospective, cross-sectional study<br><br>**Country:** Italy	**Sample:** 132 patients female: n = 88,<br>male: n = 44,<br>mean age: 51y ± 5<br><br>**Diagnostic criteria:** CSU (clinical diagnosis), unresponsive to antihistamine treatment	Clinical characteristics (activation of the coagulation/ fibrinolysis system)	**Outcome:** D-Dimer plasma levels (elevated: > 500ng/ml)<br><br>**Timepoint:** NA<br><br>**Participants included:** 132 patients female: n = 88, male: n = 44	D-Dimer were elevated among females ($p < 0.05$).<br><br>Patients with elevated D-dimer showed a significantly longer disease duration ($p < 0.01$).
Gregoriou et al. 2009<br><br>**Study name:** NA<br><br>**Design and setting:** Retrospective, observational study<br><br>**Country:** Greece	**Sample:** 2523 patients<br>female: n = 1909,<br>male: n = 614,<br>mean age: 42.1y ± 0.5<br><br>**Diagnostic criteria:** CSU (clinical diagnosis)	Clinical characteristics (etiologic aspects and prognostic factors)	**Outcome:** Incidence of etiologic aspects, prognostic factors (comorbidities, disease duration)<br><br>**Timepoint:** 2001-2006<br><br>**Participants included:** 2523 patients female: n = 1909, male: n = 614	Women had a longer disease duration of CSU compared to men ($p = 0.001$).<br><br>Women presented more often with urticaria accompanied with angioedema ($p = 0.01$).
Savic S et al. 2020<br><br>**Study name:** AWARE<br><br>**Design and setting:** Prospective, non-interventional, multi-center study	**Sample:** 252 patients female: n = 196,<br>male: n = 56,<br>mean age: 45.0 ± 15.3<br><br>**Diagnostic criteria:** CSU (clinical diagnosis)	Clinical characteristics	**Outcome:** Severity of CSU (UAS7), Quality of Life (DLQI)<br><br>**Timepoint:** 09/2014 – 08/2017<br><br>**Participants included:** 252 patients female: n = 196, male: n = 56	Severity of CSU (UAS7 score) was associated with female sex, obesity, anxiety disorder and diagnosis.<br><br>QoL (DLQI) was associated with sex (female), age, obesity, anxiety disorder and diagnosis.<br><br>Female gender was associated with anxiety disorders ($p < 0.01$).

Supplementary Table 1: Studies evaluating gender differences in urticaria

**Country:** United Kingdom				
Aktar S et al. 2015<br><br>**Study name:** NA<br><br>**Design and setting:** Case-control study<br><br>**Country:** Turkey	**Sample:** 50 patients, female: n = 32, male: n = 18, mean age: 26.40y ±8.27<br><br>**Diagnostic criteria:** CSU	Diagnostics (autologous skin or plasma serum test)	**Outcome:** Positive response of ASST/ APST was defined as at least 1.5mm diameter of erythematous papule<br><br>**Timepoint:** 04/2009-01/2010<br><br>**Participants included:** 50 patients, female: n = 32, male: n = 18	APST/ASST positivity rates were significantly higher in female subjects (p = 0.001, p = 0.032).<br><br>The diameter of the erythematous papule was remarkably larger in APST than ASST and significantly larger in females compared to males in both tests (p < 0.05).
Kolkhir P et al. 2020<br><br>**Study name:** NA<br><br>**Design and setting:** Retrospective, observational study<br><br>**Country:** Germany	**Sample:** 1613 patients,<br>female: n = 1194, male: n = 419, mean age: 43y (13-105)<br><br>**Diagnostic criteria:** CSU (clinical diagnosis)	Diagnostics (peripheral blood count)	**Outcome:** Blood count (basophils, eosinophils)<br><br>**Timepoint:** NA<br><br>**Participants included:** 1259 patients, female/male NA	Female patients with CSU had significantly lower blood eosinophile count compared with male patients with CSU (p = 0.001).
Kurt E et al. 2011<br><br>**Study name:** NA<br><br>**Design and setting:** Prospective study<br><br>**Country:** Turkey	**Sample:** 55 patients, female: n = 39, male: n = 16, mean age: 40.3y ±12.3y<br><br>**Diagnostic criteria:** CSU (clinical diagnosis)	Diagnostics (autologous serum skin test)	**Outcome:** Positive response of ASST was defined as at least 3mm diameter greater than that of negative control<br><br>**Timepoint:** NA<br><br>**Participants included:** 55 patients, female: n = 39, male: n = 16	Positive response to ASST was significantly associated with diagnosis of CSU (OR: 3.13, 95% CI: 1.25–7.87) and with female gender (OR: 3.98, 95% CI: 1.19–13.38).
Anis O et al. 2023*<br><br>**Study name:** NA<br><br>**Design and setting:** Cross-sectional, retrospective, population-based study<br><br>**Country:** Israel	**Sample:** 681 patients with interstitial cystitis/ bladder pain syndrome<br>mean age: 69y<br><br>**Diagnostic criteria:** CSU	Comorbidities (association between CSU and interstitial cystitis/ bladder pain syndrome)	**Outcome:** Prevalence of CSU in patients with interstitial cystitis/ bladder pain syndrome<br><br>**Timepoint: -**<br><br>**Participants included:** 681 patients and 3376 matched controls	The adjusted OR for CSU in patients with IC/BPS was 1.58 (95% CI 1.28-1.97). Female gender was associated with the coexistence of these disorders (OR 1.6 95% CI 1.28-2).

Supplementary Table 1: Studies evaluating gender differences in urticaria

Chen H et al. 2016<br><br>**Study name:** NA<br><br>**Design and Setting:** Cross-sectional study<br><br>**Country:** China	**Sample:** 543 patients, female: n = 378, male: n = 165, mean age: 39y<br><br>**Diagnostic criteria:** CSU (clinical diagnosis)	Comorbidities (incidence of allergic contact sensitization)	**Outcome:** Incidence of allergic contact sensitization (patch testing)<br><br>**Timepoint:** 01/2011-02/2012<br><br>**Participants included:** 543 patients, female: n = 378, male: n = 165	Among all CSU patients, 146 (62.66%) had a positive reaction to one allergen, 55 (23.61%) to two allergens, 21 (9.01%) to three allergens, 6 (2.58%) to four allergens, and only 5 (2.14%) to five or more allergens.<br><br>Positive reactions to nickel sulfate were more common among women than men (9.26% vs. 3.64%, p = 0.023), while positive reactions to potassium dichromate, benzene mix, and carba mix were more frequent among men than women (14.55% vs. 8.73%, p = 0.042; 6.67% vs. 1.85%, p = 0.004; 18.79% vs. 6.08%, p <0.001).
Confino-Cohen R et al. 2012<br><br>**Study name:** NA<br><br>**Design and Setting:** Population-based, retrospective analysis of MHS central data base<br><br>**Country:** Israel	**Sample:** 12788 patients, female: n = 8472, male: n = 4306, mean age: 45.3y ± 18.5<br><br>**Diagnostic criteria:** CSU (ICD-code)	Comorbidities (associations of CU with autoimmune diseases)	**Outcome:** Prevalence of comorbidities<br><br>**Timepoint:** 01/1993 – 03/2010<br><br>**Participants included:** 12788 patients, female: n = 8472, male: n = 4306	For male and female patients, having CSU was associated with an increased odds ratio for hypothyroidism (p < 0.0005), hyperthyroidism (p < 0.0005) and antithyroid antibodies (p < 0.0005).<br><br>Female patients with CU had a significantly higher incidence of rheumatoid arthritis (p < 0.0005), Sjögren syndrome (p < 0.0005), celiac disease (p < 0.0005), type I diabetes mellitus (p < 0.0005), and systemic lupus erythematosus (p < 0.0005), mostly diagnosed during the 10 years after the diagnosis of CU.
Chiu H et al. 2018<br><br>**Study name:** NA<br><br>**Design and setting:** Population-based retrospective cohort study<br><br>**Country**: Taiwan	**Sample:** 9332 patients<br>female: n = 5667,<br>male: n = 3665,<br>mean age: 37.7y ± 17.6<br><br>**Diagnostic criteria:** CSU (ICD-code)	Comorbidities (associations of CU with atopic and autoimmune diseases)	**Outcome:** Prevalence of comorbidities<br><br>**Timepoint:** 01/1996-12/2013<br><br>**Participants included:** 46660 patients/ controls<br>female: n = 28335<br>male: n = 18325	Comorbidities among women:<br>atopic dermatitis (OR 1.97), allergic rhinitis (OR 1.49), autoimmune thyroid diseases (OR 1.31), systemic lupus erythematodes (OR 1.53), vitiligo (OR 1.65), Hennoch-Schönlein purpura (OR 2.46)<br><br>Comorbidities among men:<br>atopic dermatitis (OR 1.89), allergic rhinitis (OR 1.57), autoimmune thyroid diseases (OR 1.36), Kawasaki disease (OR 2.99), inflammatory bowel disease (OR 1.41)
Ghazanfar M et al. 2020	**Sample:** 12185 patients<br>female: n = 8352,	Comorbidities	**Outcome:** Prevalence of comorbidities<br><br>**Timepoint:** 01/1994-12/2015	There were more female patients with CSU compared to males (69% vs 32%).

Supplementary Table 1: Studies evaluating gender differences in urticaria

**Study name:** NA<br><br>**Design and setting:** Retrospective study<br><br>**Country**: Denmark	male: n = 3833,<br>mean age: 38.4y<br><br>**Diagnostic criteria:** CSU (ICD-code)		**Participants included:**<br>12185 patients<br>female: n = 8352,<br>male: n = 3833,	The most common comorbidities in the CU group, when counting at the time of diagnosis and during follow up were depressions (4.4%), rhino-conjunctivitis (2.9%), osteoporosis (2.9%), atopic dermatitis (2.5%), and diabetes mellitus (2.3%), while the least common were peptic ulcers (0.1%), psychosis (1.0%), anaphylaxis (0.6%), thyroiditis (0.3%), SLE (0.3%), and vitiligo (0.1%).<br><br>There was a significantly higher OR of thyroiditis and vitiligo among women than men, OR = 1.45 (2.79–6.72) and OR = 9.75 (2.8–35.15).
Gupta P et al 2023<br><br>**Study name:** NA<br><br>**Design and setting:** Cross-sectional, observational, single center<br><br>**Country**: India	**Sample:** 481 patients<br>female: n = 261,<br>male: n = 220,<br>mean age: 33.41y ± 10.6<br><br>**Diagnostic criteria:** CSU (clinical diagnosis)	Comorbidities (association with metabolic syndrome)	**Outcome:** Prevalence of metabolic syndrome<br><br>**Timepoint:** NA<br><br>**Participants included:**<br>481 patients, female: n = 261,<br>male: n = 220,	Males with CSU were more likely to have impaired glucose tolerance compared to controls (p = 0.022), while females with CSU had a higher prevalence of central obesity (p = 0.015).
Kolkhir P et al. 2021<br><br>**Study name:** NA<br><br>**Design and setting:** Retrospective, observational study<br><br>**Country**: Germany	**Sample:** 1199 patients<br>female: NA,<br>male: NA,<br>mean age: NA<br><br>**Diagnostic criteria:** CSU (clinical diagnosis)	Comorbidities (autoimmune diseases), treatment (omalizumab)	**Outcome:** Prevalence of autoimmune diseases, response to treatment (complete response: reduction of UAS7 0f 90%)<br><br>**Timepoint:** NA<br><br>**Participants included:**<br>1199 patients	Autoimmune comorbidities were reported more frequently in female CSU patients (OR = 2.3, P < 0.001).
Grieco T et al. 2020<br><br>**Study name:** NA<br><br>**Design and setting:** Prospective,	**Sample:** 42 patients<br>female: n = 39,<br>male: n = 15<br>mean age: 48.89y ± 18.72	Treatment (identifying differences between male and female patients receiving omalizumab in terms of non-response)	**Outcome:** Assessing the change rate of the UAS from baseline, dosage of the treatment<br><br>**Timepoint:** 09/2016-10/2019	Female sex was associated to a significantly higher frequency of recurrence (77.4% in respect to male sex 36.4%, p = 0.024).

observational, monocentric study<br><br>**Country**: Italy	**Diagnostic criteria:** CSU		**Participants included:**<br>42 patients, female: n = 39, male: n = 15	
Kocaturk E et al.<br><br>**Study name:** NA<br><br>**Design and setting** Retrospective, observational study<br><br>**Country**: Turkey	**Sample:** n = 110 patients<br>male: n = 36<br>female: n = 74<br>mean age: 40.98 ± 12.37y<br><br>**Diagnostic criteria:** CSU (clinical diagnosis)	Treatment (omalizumab, cyclosporin A)	**Outcome:** OS of treatment response by UCT > 12 = under control<br><br>**Timepoint**: NA<br><br>**Participants included:** 110 patients (36 male, 74 female)	Female gender was more frequent in non-responders towards CsA and Omalizumab (p = 0.017).
Tagka A et al. 2021<br><br>**Study name:** NA<br><br>**Design and setting:** Prospective, observational study<br><br>**Country**: Greece	**Sample:** 108 patients<br>female: n = 83,<br>male: n = 25,<br>mean age: 47.83±16.24<br><br>**Diagnostic criteria:** CSU according to clinical examination	Treatment (identifying differences between male and female patients receiving omalizumab in terms of clinical efficacy)	**Outcome:** Time to relapse<br><br>**Timepoint:** 2014-2016<br><br>**Participants included:**<br>108 patients<br>female: n = 83,<br>male: n = 25	Males manifested a mean CSU score 29.26±9.74 at diagnosis, whereas females manifested a mean CSU score 34.19±8.96 at diagnosis (p=0.024). In addition, the mean CSU score was 9.00±6.97 for males and 14.45±11.47 for females. The mean CSU score of the first trimester was 14.85±11.43 for males and 20.77±10.79 for females. Finally, the mean CSU score of the first semester was 11.35±10.40 for males and 16.26±10.68 for females.<br><br>Females manifested lower levels of percentage change both with respect to the initial CSU score at diagnosis (p= 0.03).
Sirufo M et al. 2021<br><br>**Study name:** NA<br><br>**Design and setting:** Prospective, non-interventional study<br><br>**Country**: Italy	**Sample:** 42 patients<br>female: n = 26, mean age: 48y ± 11.3<br>male: n = 16, mean age: 48.1y ± 22.3<br><br>**Diagnostic criteria:** CSU > 3 months, resistant to H1-AH	Treatment (identifying differences between male and female patients receiving omalizumab in terms of tolerability, safety, and clinical efficacy)	**Outcome:** DLQI, UAS and CU-Q2oL were used to assess disease activity and guide assessment of therapy effectiveness<br><br>**Timepoint**: 01/2017-11/2019 (25 months Follow-Up)<br><br>**Participants included:**	All patients, regardless of sex, age or any other factor achieved the clinical remission of the disease after the first 3 doses with a reduction of disease activity indices and impact on the quality of life (UAS/ DLQI/ CU-Q2oL: 0 points for both gender at the end of the treatment).

Supplementary Table 1: Studies evaluating gender differences in urticaria

			42 patients, female: n = 26, male: n = 16	Recurrence rates were predominantly in men: 25% recovered after the first cycle, in females 84.6% were in complete remission.<br><br>Women had a higher ASST positive rate (30.8% vs. 25%).
Sommer R et al. 2020<br><br>**Study name:** NA<br><br>**Design and setting:** Cross-sectional, non-interventional study<br><br>**Country:** Germany	**Sample:** 103 patients female: n = 75, male: n = 29, mean age: 43.9y ± 14.9<br><br>**Diagnostic criteria:** CSU (clinical diagnosis)	Treatment (patient burden and needs in treatment)	**Outcome:** DLQI, CU-Q2oL, Patient Needs Questionnaire<br><br>**Timepoint**: 2009-2011<br><br>**Participants included:** 103 patients, female: n = 74, male: n = 29	Differences in patient needs were found for the needs "to be able to accept oneself" (mean PNQ score: 2.83 for women vs. 1.85 for men, z = -2.38, p = 0.017), "to find a clear diagnosis and therapy" (3.86 for women vs. 3.43 for men, z = -2.70, p = 0.007), "to be healed of all skin defects" (3.78 for women vs. 3.41 for men, z = -2.81, p = 0.005), and "to be less helpless against the disease" (3.66 for women vs. 3.39 for men, z = -2.32, p = 0.021).
De Ue et al. 2011<br><br>**Study name:** NA<br><br>**Design and setting:** Cross-sectional, non-interventional study<br><br>**Country**: Brazil	**Sample:** 62 patients female: n = 45, male: n = 17, mean age: 39.8y ± 12<br><br>**Diagnostic criteria:** CSU (clinical diagnosis)	Quality of life	**Outcome:** DLQI, SF-36<br><br>**Timepoint**: NA<br><br>**Participants included:** 62 patients, female: n = 45, male: n = 17	Quality of life was found to be more affected in women, with statistically significant differences com-pared to men in the daily activities domain of the DLQI (p=0.003) and in the vitality (p=0.038), role-emotional (p=0.018) and mental health (p=0.020) domains of the SF-36
Erol K et al. 2020<br><br>**Study name:** NA<br><br>**Design and setting:** Cross-sectional, non-interventional study<br><br>**Country**: Turkey	**Sample:** 103 patients female: n = 76, male: n = 27, mean age: 40.62y ± 12.51<br><br>**Diagnostic criteria:** CSU (clinical diagnosis)	Quality of life (fatigue)	**Outcome:** DLQI, HADS, FSS<br><br>**Timepoint**: 12/2018 – 02/2019<br><br>**Participants included:** 103 patients, male n = 27, female: n = 76	Female patients showed more fatigue than males and had significantly higher scores pf FSS (p = 0.025).<br><br>Whereas 42 of 76 (55.3%) female patients with CSU had fatigue, only 8 of 27 (29.6%) males had fatigue (p = 0.022)

Supplementary Table 1: Studies evaluating gender differences in urticaria

Sanchez-Diaz et al. 2023<br><br>**Study name:** NA<br><br>**Design and setting:** Cross-sectional, non-interventional study<br><br>**Country:** Spain	**Sample:** 77 patients female: n = 53, male: n = 22, mean age: 46.48y ± 11.25<br><br>**Diagnostic criteria:** CSU (clinical diagnosis)	Quality of life	**Outcome:** DLQI, CU-Q2oL, International Index of Erectile Function (IIEF-5), Female Sexual Function Index (FSFI-6), NRS for sexual impairment<br><br>**Timepoint**: 01/2020 – 08/2021<br><br>**Participants included:** 77 patients, female: n = 53, male: n = 22	Female sex was significantly associated with poorer general dermatologic and urticaria-specific quality of life ($p < 0.05$).<br><br>Female sex, and the presence of sexual dysfunction were confirmed to be strongly related to poor quality of life ($p < 0.01$) measured both by DLQI and CU-Q2oL<br><br>Female sexual dysfunction was associated with an increase in the risk for anxiety by 85% (OR: 1.85, CI: 1.03–3.30, p = 0.002), depression by 90% (OR: 1.90, CI: 1.02–3.88, p = 0.04) and sleep disturbances ($p < 0.001$).<br><br>Male sexual dysfunction was not related to DLQI, CU-Q2oL, anxiety, depression or sleep disturbances.
Silvares M et al. 2011<br><br>**Study name:** NA<br><br>**Design and setting:** Cross-sectional, non-interventional study<br><br>**Country:** Brazil	**Sample:** 100 patients, female: n = 86, male: n = 14, mean age: 41.8y ±14.5<br><br>**Diagnostic criteria:** clinical diagnosis of CSU	Quality of life	**Outcome:** DLQI<br><br>**Timepoint**: 05/2009-03/2010<br><br>**Participants included:** 100 patients, male n = 14, female: n = 86,	The mean total DLQI as "severe impairment of quality of life" was classified by a female predominance (6:1).<br><br>There was a greater impact of CU on quality of life of women in question 4 (clothing), while in question 7 (work/study) and question 10 (treatment), the impact was greater for men ($p < 0.05$).

* only abstract available

ASST: autologous serum skin test; CI: confidence interval; CsA: cyclosporin A; CSU: chronic spontaneous urticara; CU-Q2oL: chronic urticaria quality of life test; DLQI: Dermatology Life Quality Index; FSFI-6: Female Sexual Function Index; FSS: fatigue severity scale; HADS: hospital anxiety and depression scale; IIEF-5: International Index of Erectile Function; OR: odds ratio; SF-36: short form 36; UAS7: urticaria severity score; UCT: urticaria control test
